# Stimulus devaluation induced by action stopping is greater for explicit value representations

**DOI:** 10.3389/fpsyg.2015.01640

**Published:** 2015-10-28

**Authors:** Jan R. Wessel, Alexandra L. Tonnesen, Adam R. Aron

**Affiliations:** ^1^Department of Psychology, University of California, San Diego, CA, USA; ^2^Department of Psychological and Brain Sciences, College of Liberal Arts and Sciences, University of Iowa, Iowa City, IA, USA; ^3^Department of Neurology, Carver College of Medicine, University of Iowa, Iowa City, IA, USA

**Keywords:** value, stop-signal task, inhibitory control, implicit learning, devaluation, cognitive control

## Abstract

We recently showed that rapidly stopping an action in the face of a reward-related stimulus reduces the subjective value of that stimulus (Wessel et al., 2014). In that study, there were three phases. In an initial learning phase, geometric shapes were associated with monetary value via implicit learning. In a subsequent treatment phase, half the shapes were paired with action stopping, and half were not. In a final auction phase, shapes that had been paired with stopping in the treatment phase were subjectively perceived as less valuable compared to those that were not. Exploratory *post hoc* analyses showed that the stopping-induced devaluation effect was larger for participants with greater explicit knowledge of stimulus values. Here, we repeated the study in 65 participants to systematically test whether the level of explicit knowledge influences the degree of devaluation. The results replicated the core result that action stopping reduces stimulus value. Furthermore, they showed that this effect was indeed significantly larger in participants with more explicit knowledge of the relative stimulus values in the learning phase. These results speak to the robustness of the stopping-induced devaluation effect, and furthermore imply that behavioral therapies using stopping could be successful in devaluing real-world stimuli, insofar as stimulus values are explicitly represented. Finally, to facilitate future investigations into the applicability of these findings, as well as the mechanisms underlying stopping-induced stimulus devaluation, we herein provide open source code for the behavioral paradigm.

## Introduction

Overvaluation of reward-associated stimuli is a common feature of many unhealthy and maladaptive behaviors. For example, obese people show an increased response in value-associated brain regions when viewing pictures of high-caloric foods (Rothemund et al., 2007), smoking-related cues increase nicotine consumption and craving in smokers (Hogarth et al., 2010), and monetary cues evoke disproportionate value-related neural activity in pathological gamblers (Clark et al., 2013; Sescousse et al., 2013). Hence, changing such valuation patterns could be key in enabling people to overcome unhealthy behaviors triggered by external stimuli (Wisotsky and Swencionis, 2003; Root et al., 2009; Rupprecht et al., 2015).

One potential way to reduce the value of a stimulus is through motor inhibition. Several authors have hypothesized that stopping or withholding a response in the face of a valuable stimulus might lead to devaluation of that stimulus (Veling et al., 2008, 2013; Houben et al., 2012a). Many studies have been conducted to test whether stopping/withholding a response can indeed lead to behavioral changes that could potentially be reflective of stimulus devaluation. Such studies used stimuli that ranged from food (Nederkoorn et al., 2010, 2012; Houben, 2011; Houben and Jansen, 2011; Houben et al., 2012b; Allom and Mullan, 2015; Lawrence et al., 2015) to emotional pictures and faces (Fenske et al., 2005; Raymond et al., 2005; Kiss et al., 2008; Veling et al., 2008; Doallo et al., 2012; Ferrey et al., 2012; Frischen et al., 2012) to alcoholic beverages (Houben et al., 2011, 2012a; Bowley et al., 2013; Jones and Field, 2013). However, as we have argued previously (Wessel et al., 2014), not all of these studies operationalized response inhibition in a way that actually requires the stopping of an initiated response, and not all measured value in a way that is economically sound (contrast that with Schonberg et al., 2011, 2014).

In our recent report, we presented an experimental paradigm that *does* require rapid action stopping of an initiated response, and *does* employ an economically sound measure of subjective value (Wessel et al., 2014). Using this paradigm, we showed that rapid action stopping in the face of rewarding stimuli reduces the subjective value of those stimuli. The behavioral paradigm had three phases. First, we associated different geometric shapes (triangles, circles, etc.) with monetary reward. In this initial learning phase, participants were instructed to respond to the appearance of a given shape on each trial, with a reward being delivered after each response. Unbeknownst to the participants, reward delivery followed a fixed schedule, which associated each shape with a specific value. In a subsequent treatment phase, participants then performed a version of the stop-signal task (Logan et al., 1984) in which they responded to the same shapes that were presented to them in the learning phase. Half of the shapes were sometimes paired with stop signals, while the other half of the shapes was never paired with stopping. Finally, we probed participants’ subjective valuations of the shapes using an auction procedure from behavioral economics (Becker et al., 1964). We found that the shapes that had been previously paired with action stopping were perceived as less valuable.

Notably, that study was designed to test the devaluation of largely *implicit* value associations: participants were not supposed to know that the shapes were differentially rewarded in the learning phase, since we conjectured that implicit value representations could be more susceptible to behavioral change than explicit representations (Marteau et al., 2012). A post-experimental questionnaire, which assessed participants’ explicit awareness of the fixed reward schedule in the learning phase by probing their verbalizable knowledge (Seger, 1994), showed that our procedure largely achieved its aim of associating shape-value at an implicit level: only 15 out of 55 participants reported noticing a contingency. However, an exploratory data analysis showed that—contrary to the initial assumption— this subsample of 15 participants showed even greater stopping-induced stimulus devaluation (i.e., an increased effect size compared to the subgroup without any explicit knowledge). However, this observation was not the result of a systematic investigation, and furthermore resulted from a numerical comparison of the effect sizes rather than a direct test.

The question of whether conscious awareness of one’s subjective valuations affects stopping-induced stimulus devaluation is important, since it could have implications for which kinds of real-world stimuli (cigarettes, cake, alcohol) could be devalued, and how such devaluation could be optimally achieved. Hence, we here aimed to systematically test whether stopping-induced stimulus devaluation differentially affects explicit or implicit value representations. We used the same paradigm as in our previous study, in a large sample of 65 participants. We aimed to categorize participants into two separate groups, based on their verbalizable knowledge of the stimulus values, and to compare these groups with respect to their stopping-induced stimulus devaluation. Based on our previous exploratory analysis (Wessel et al., 2014; see above), we predicted that the group of participants with explicit knowledge of the reward contingency would show increased stopping-induced stimulus valuation compared to the group with no such knowledge.

## Materials and Methods

### Participants

Sixty-seven healthy volunteers were recruited using flyers on the UCSD campus, and were paid for participation ($10 base-payment, additional money to be earned in the task). Participants signed written informed consent prior to participating in the study, the study was approved by the local institutional review board, and all experimentation was conducted in accordance with the Declaration of Helsinki. Two participants were excluded based on their stop signal task data (see below for exclusion criteria), leaving a sample of *N* = 65 (mean age 21.54 years, range: 18–53 year; 36 female, one left-handed).

### Materials

Stimuli were presented on Apple Macintosh computers (Apple Inc., Cupertino, CA, USA) running MATLAB 2012a (The MathWorks, Natick, MA, USA) and Psychtoolbox 3 (Brainard, 1997). Responses were made using a QWERTY-keyboard.

### Experimental Task

The experiment was identical to Experiment 2 of our previous study (Wessel et al., 2014), with the exception that the number of trials varied for part of this sample (see below). Accordingly, below we mostly recapitulate the very methods section from that paper (Wessel et al., 2014, p. 2317–2320). We also provide the MATLAB code for the experiment, along with detailed documentation on how to run it at https://github.com/janwessel/stopdeval.

**FIGURE 1:**
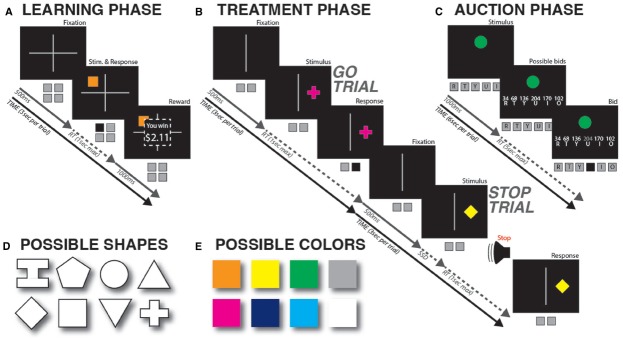
**Task diagram. (A)** Learning phase. Each shape was repeatedly shown and rewarded according to a pre-defined schedule that associated it with one of four different reward values. **(B)** Treatment phase. One of the two shapes per value level was paired with stop-signals 75% of the time, while the other was never paired with stopping. **(C)** Auction phase. Participants repeatedly bid monetary value on each of the shapes from the previous two phases. **(D,E)** Possible shapes and colors. Colors and shapes were randomly paired for each participant, and the resultant combination was then randomly assigned to one of the four value levels in the learning phase, as well as either the stopping or non-stopping condition in the treatment phase.

The task was divided into three parts (Figures 1A–C).

#### Learning Phase

In this phase (Figure 1A), we associated eight different geometric shapes with four different monetary values. Two shapes each were associated with mean values of $0.5, $1, $2, or $4, respectively. The shapes were a square, a circle, a diamond, a triangle, an inverted triangle, a cross, a hexagon, and an “I”-shape (Figure 1D). The different shapes were also colored (white, green, blue, yellow, cyan, magenta, orange, or gray; Figure 1E), in order to maximize the chances of acquiring an implicit value association. Color-shape pairings were randomized for each participant, and then remained constant throughout the experiment. The shapes were randomly assigned to a given value for each participant.

A trial proceeded as follows. A fixation cross was presented for 500 ms, which divided the screen into four quadrants. Then, one of the eight shapes appeared in one of the quadrants. Participants pressed one button (out of four possible) corresponding to that quadrant. There was a deadline of 1000 ms; if no response was made within that period, a “too slow” message appeared for 1000 ms. Immediately after the response, a payment amount was indicated on the screen: a black square was superimposed on the stimulus display, within which the message “You won $ X.XX” appeared (for 1000 ms). Participants were told that responding “as fast and accurately as possible” would lead to higher rewards. To ensure that participants paid attention to the magnitude of reward in this phase, we told them that the computer would randomly pick five of the trials at the end of the experiment and pay out the amount associated with those trials. After the reward display, the screen was cleared before the next trial, resulting in a trial duration of 3000 ms. Trials on which misses (no response before deadline) or errors (wrong button) occurred were repeated until the correct response was made. Most importantly, unbeknownst to the participants, the trial-by-trial monetary reward schedule was actually independent of performance, and instead followed a pre-defined schedule: Each stimulus had one of four fixed distributions of possible payout values randomly assigned to them. The distributions centered around four different means (either $0.5, $1, $2, or $4), with a uniform dispersion of ±25c around those means. To ensure that the value representations were largely implicit, only 80% of the trials per shape were rewarded according to this distribution. On the remaining 20% of trials, the reward was 0^1^. Each shape had an identical probability of appearing in any of the four quadrants. Thirty-one participants performed 50 trials per shape (400 overall), which were presented in four blocks.

After running 31 participants, we paused and assessed the number of participants with at least partially explicit knowledge (see below for procedure). As only five of these had any explicit knowledge, we increased the number of trials in the learning phase for the remaining sample of participants (*N* = 34). These participants performed 72 trials per shape (60 rewarded according to the schedule, 12 non-rewarded), which were presented in six blocks. We reasoned that more value training would increase the number of participants with explicit value representations; better allowing us to compare groups with and without explicit value representations.

#### Treatment Phase

In this phase (Figure 1B), we used the eight shapes from the earlier learning phase, which were now associated with different values. The primary task for the participants was to make a quick motor response according to the placement of the shape on the computer screen (left or right), and to stop their impending response when a stop-signal occurred. Four out of these eight shapes (one per value-step, i.e., one shape each associated with a mean value of $0.5, $1, $2, or $4) were always paired with Go trials (i.e., a stop signal was never presented on trials with these shapes). The other half of the eight shapes (which were also associated with mean values of $0.5, $1, $2, or $4), were paired with stop signals on some of the trials (on 75% of trials for each stimulus; i.e., 27 out of 36 trials). Ideally, these latter shapes would be paired 100% of the time with stop signals (to increase the potential effect of action stopping on stimulus valuation). However, a relative probability of stop vs. go-trials of 0.5 would diminish the prepotency of the go-response. Furthermore, we were concerned that a fully deterministic pairing of some shapes with stopping on 100% of trials would potentially lead to the emergence of explicit knowledge on the participants’ part. Such awareness of the pairing might cause participants to consciously withhold their response on these shapes on trials after they picked up on the contingency, which would turn this task into a decision making task instead of a stopping-task (because participants might just decide to never initiate a response once they realize that some shapes are always paired with stopping). Hence, we decided to pair the stopping-shapes with stopping on 75% of the trials only (resulting in an overall probability of a stop-signal of 37.5%), in order to achieve a good tradeoff between maximizing the potential effects of stopping on devaluation and avoiding the detrimental effect of a deterministic contingency.

A trial proceeded as follows. The screen was divided into two halves by a vertical line for 500 ms. The same shapes from the learning phase then appeared to the left or right of this line. Participants were instructed to respond as fast and accurately as possible according to the position of the shape by using one of two buttons on the keyboard (deadline: 1000 ms), one with their left hand, and one with their right hand. Participants were instructed that occasional stop-signals (200 ms sine-wave tones, 900 Hz) would occur shortly after stimulus-onset—in which case they should try to cancel the response. The stop-signal delay (SSD) was adapted separately for left and right responses depending on ongoing performance (+50 ms following successful stop-trials, –50 ms following failed stop-trials) to achieve an overall probability of successful stopping p(stop) of 0.5 (Verbruggen and Logan, 2009). The SSD’s initial value was set to 250 ms. Participants were instructed that successful stopping on stop-trials and fast responding on go-trials were equally important. The first 31 participants performed 36 trials for each shape (288 overall), split into eight blocks. The remaining 34 participants, for whom we changed the trial numbers in the learning phase, performed 44 trials for each shape (352 overall), split into 11 blocks. In the breaks between blocks, participants received information about their Go-trial reaction time (GoRT), as well as their miss- and (direction-) error-rates. Additionally, the experimenter (but not the participant) received information about p(stop) and SSD to ensure that participants were not overly favoring stopping over going or *vice versa*. In case the participant appeared to favor either strategy, the experimenter informed the participant to remember that both stopping and going were equally important. We aimed to achieve the following behavioral parameters in each block: GoRT between 400 and 650 ms, p(stop) between 0.4 and 0.6, and SSD > 100 ms. Note that in this treatment phase subjects were not reimbursed—in effect, the cues were presented in extinction.

#### Valuation Phase

In this phase (Figure 1C), we repeatedly presented each of the eight shapes (the same ones as those from the earlier two phases) to the participants and instructed them to bid money on each shape according to an auction procedure (Becker et al., 1964) designed to assess the “true” subjective value of a given good or object. The participants were presented with six different cent-amounts that they could choose as their bid using one of six keys on the keyboard (r, t, y, u, i, o; the button-mappings were spatially congruent and displayed on the screen under the respective values). Participants were instructed to choose the cent-amount that most closely represented their subjective valuation of the shape. This was implemented using an auction procedure (the exact instructions can be found along with the MATLAB code). Participants were instructed to neither overbid nor underbid, as both of those strategies would be suboptimal. They were told that optimal bidding behavior would lead to larger payoffs, and that the payoff from the auction phase would be paid out at the end of the experiment, in addition to the payment from the initial learning phase.

A trial proceeded as follows. A fixation-cross was displayed for 500 ms, after which one of the shapes from the earlier two phases was presented centrally for 1,500 ms. Then, the six potential bids and their button mappings were presented, and participants had 5,000 ms to pick an amount to bid. The overall trial-duration was fixed at 6,000 ms, with the remainder of the 5,000 ms response-window going to the inter-trial interval. Each of the eight shapes was presented 10 times (i.e., there were 80 trials total), each time with six potentials bids, as explained above. These bids came from five different sets of values. Specifically: (34, 68, 102, 136, 170, 204), (39, 78, 117, 156, 195, 234), (44, 88, 132, 176, 220, 264), (49, 98, 147, 196, 245, 294), and (54, 108, 162, 216, 270, 324). We chose to use multiple different sets of values in order to induce variance into the bidding behavior, specifically, so that participants did not bid the exact same value every time they saw a given shape. These sets of bids were chosen so that the overall range of values completely covered the range of true values that were associated with each shape in the learning phase (40, 80, 160, and 320 cents, respectively). Each set was presented twice for each shape, in random order. The bids within each set were randomly assigned to one of the six response buttons on each trial. The shapes themselves were also presented in random order across the 80 trials. Trials were presented in four blocks.

## Procedure

Participants were first instructed on both the learning-phase and the treatment-phase. They practiced both phases briefly (10 trials each), before performing the actual learning- and treatment-phases. Thereafter, they read the instructions of the auction. The experimenter made sure that the auction procedure was fully understood, placing special emphasis on the fact that systematic over- or underbidding, or bidding according to any other rationale than the participant’s subjective value, would lead to suboptimal earnings at the end of the experiment. After the valuation-phase, we assessed participants’ conscious awareness of both the learning phase and stopping phase regularities using the questionnaire described in the next paragraph. Finally, the computer picked five trials from the learning phase, which were added and paid out to the participants, in addition to a $2 bonus for the valuation phase (while participants were bidding with the expectation that five random trials would be paid out, we did not actually perform an artificial auction, and instead paid this constant amount for simplicity). Participants came away from the experiment with between $4 and $14 in addition to the base payment of $10.

### Probe of Explicit Awareness of Relative Stimulus Values

We used a questionnaire to verbally probe the amount of explicit knowledge about the two main regularities of the procedure: (1) the reward schedule in the learning phase (question 1 below) and (2) the fact that only some shapes were paired with stop-signals in the stopping phase (question 2 below). The experimenter asked the participants the following five questions (numbering is for the reader and was not provided to participants):

  (1a) What strategy did you use in the last part of the experiment? (auction phase)
  (1b) Did you think that any shapes were associated with higher or lower reward during the first phase of the experiment? (learning phase)
  (1c) If so, which? If so, did you utilize that information in the last phase? (auction phase)
  (2a) Did you notice anything about the second phase of the experiment? (stopping phase)
  (2b) Did you notice that some shapes were paired with stop signals more often than others? If so, which?
(3) Any other comments or remarks?

### Analysis

#### Types of Knowledge

The questionnaire assayed the level of verbalizable explicit knowledge of the reward schedule in the learning phase. The classification was done as follows.

***Explicit learner***

This designation was given if a participant reported a “feeling of knowing” of the regularities in response to the open question about the auction phase (1a), and could accurately name at least one shape for which the regularity was true in response to question 1b. For example, the participant could state “The green square was always paired with high reward,” or “The white diamond was always paired with low rewards.”

***Implicit learner***

These participants reported not noticing any regularities in response to any of the questions in the debriefing questionnaire.

#### Valuation Phase

The effects of interest were measured in the auction phase. They were tested using a 2 × 2 × 4 ANOVA with the within-subjects factors VALUE (four levels: low, medium low, medium high, high) and STOPPING (two levels: 0 and 75%), the between-subjects factor KNOWLEDGE (two levels: explicit, implicit), and the dependent variable BIDDING-LEVEL (1–6, ranging from the lowest to the highest option within each set of bids). Before parametric testing, the main variable of interest (the devaluation score—i.e., the difference score of all stop-trial bids and all go-trials bids) was tested for outliers using the 1.5 × interquartile range criterion for each group (explicit and implicit) separately. If any subjects were excluded from the parametric tests based on this criterion, testing was repeated using a non-parametric test for which the outliers were included. Effect sizes for the ANOVA were expressed in units of partial η2 (denoted ${\text{η}}_{p}^{2}$), and in units of *r* [with *r* = *Z*/sqrt(*N*)] for the non-parametric tests.

#### Treatment Phase

To ensure the validity of the race model and to verify that stopping behavior was typical, we also analyzed the behavioral data from the stopping phase. To examine the validity of the race model, we tested whether GoRT was slower than failed-stop RT for each participant. We also examined the data in terms of the probability of stopping (which should be in the range 0.4–0.6) and stop-signal reaction time (SSRT), which should be in the range of about 120–300 ms, based on typical manual stopping paradigms using auditory stop-signals. Two participants that did not fulfill these criteria were excluded from the sample. We calculated SSRT using the mean method (Verbruggen and Logan, 2009).

## Results

### Treatment Phase

Mean GoRT was 467 ms (SEM = 7.5 ms), failed-stop RT was 393 ms (SEM = 6.3 ms). This difference was significant [*t*(64) = 23.7, *p* = 2.1 × 10^–33^, *d* = 1.35], validating the independence assumption of the race model. Error (0.2%) and miss rates (0.6%) were low. Mean p(stop) was 0.52 (SEM = 0.002), SSD was 279 ms (SEM = 8.5 ms), and SSRT was 188 ms (SEM = 3.7 ms).

### Types of Knowledge

Overall, 22 out of the 65 participants were classified as explicit learners, i.e., they noted that some of the shapes in the learning phase were systematically rewarded with higher or lower amounts, and could name the relative value of at least one of the shapes. The remaining 43 participants showed no verbalizable knowledge of the regularity [neither unprompted (question 1a) nor prompted (question 1b)] and were hence classified as implicit learners.

### Valuation Phase

**FIGURE 2:**
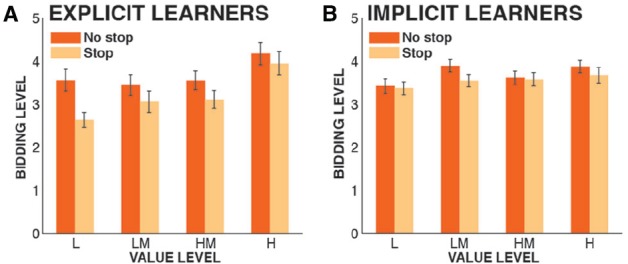
**Bidding data from the valuation phase, split by group.** Depicted are bidding levels from low (1) to high (6), plotted by the actual value of each shape from the learning phase (L, low; LM, low-medium; HM, high-medium; H, high). Error bars denote the standard error of the mean across subjects. **(A)** Explicit learners; **(B)** Implicit learners.

Overall, six outliers were removed from the sample (based on the abovementioned 1.5 × IQR criterion for the devaluation score) before parametric testing, leaving 59 participants in the analysis. The 2 × 2 × 4 ANOVA revealed a significant main effect of VALUE [*F*(3,171) = 10.23, *p* < 0.0001, ${\text{η}}_{p}^{2}$ = 0.152], showing that participants bid significantly higher amounts on shapes of higher value^2^. The main effect of STOPPING was also significant [*F*(1,57) = 9.74, *p* = 0.0028, ${\text{η}}_{p}^{2}$ = 0.146], replicating our previous finding that participants bid significantly less for shapes that were paired with stopping compared to shapes that were not. There was no main effect of KNOWLEDGE [*F*(1,57) = 1.5, *p* = 0.23, ${\text{η}}_{p}^{2}$ = 0.026], meaning that participants did not bid significantly higher or lower overall amounts on the shapes based on whether they had explicit or implicit knowledge of the regularity. Crucially, regarding our main prediction, there was a significant KNOWLEDGE × STOPPING interaction [*F*(1,57) = 13.9, *p* = 0.0004, ${\text{η}}_{p}^{2}$ = 0.196; Figure 2], indicating that explicit learners showed significantly increased stopping-induced stimulus devaluation. Furthermore, there was a significant KNOWLEDGE × VALUE interaction [*F*(3,171) = 4.3, *p* = 0.0059, ${\text{η}}_{p}^{2}$ = 0.072], showing that explicit learners bid higher amounts for shapes with relatively higher value (and *vice versa*) compared to implicit learners, reflecting the fact that they had a better representation of the relative differential values of the shapes. Finally, there was no significant VALUE × STOPPING interaction [*F*(3,171) = 0.95, *p* = 0.42, ${\text{η}}_{p}^{2}$ = 0.038], and no significant three-way interaction [*F*(3,171) = 0.88, *p* = 0.45 ${\text{η}}_{p}^{2}$ = 0.012].

### Within-Subjects Analysis

**FIGURE 3:**
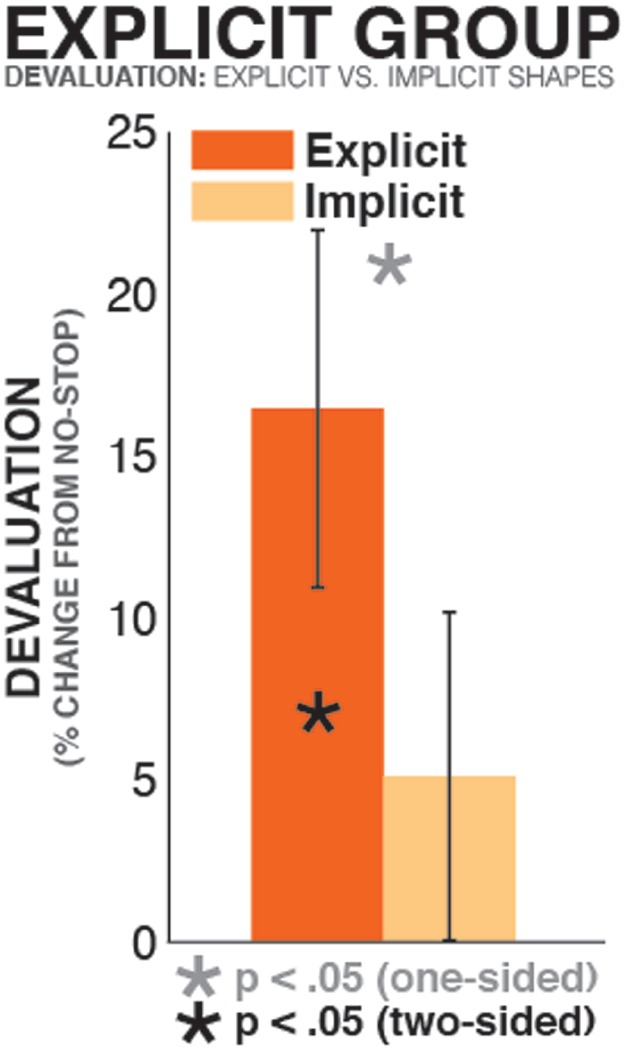
**Devaluation in the explicit learner group, split by type of knowledge.** The bars show stopping-induced devaluation (% reduction of value on stop-shapes compared to non-stop shapes), separately for pairs of stimuli of same value in which (a) the value of at least one of the two (stop shape or no-stop shape) was explicitly represented (orange bar), or (b) the value of neither shape was explicitly represented (yellow bar).

To investigate whether greater stopping-induced devaluation in the explicit learner group was indeed due to their explicit representation of the value of a subset of shapes, we directly compared the degree of stopping-induced devaluation between explicitly and implicitly represented shapes in the explicit learner group. To this end, in each explicit learner, we quantified stopping-induced stimulus devaluation (in units of percentual bidding level reduction on shapes that were paired with stopping relative to shapes that were not paired with stopping) separately for individual pairs of shapes of identical value. Each pair of shapes (i.e., the two $0.5 shapes, the two $1 shapes, and so on) was classified by whether the value of at least one of the two shapes (the one paired with stopping or the one not paired with stopping) was explicitly represented, or whether both shapes’ value was implicit (i.e., neither shape was named in the post-experimental questionnaire as being of relatively high, low, or medium value). In cases in which more than one pair of shapes fit either criterion in a given participant, the median of values was taken. These values were then converted into units of percentual change between the shape paired with stopping and the shape not paired with stopping (see above) for each individual participant, and tested against each other using a paired samples *t*-test. Participants in which all four pairs of shapes were classified in the same condition (viz., at least one shape in each of the four steps of value had explicitly represented value) were excluded from this analysis, leaving a final sample of *N* = 16. This analysis showed that there was significant stopping-induced devaluation for pairs of shapes in which the value of at least one of the two was explicitly represented [*t*(15) = 2.76, *p* = 0.015, *d* = 0.88]. However, in pairs of stimuli in which neither shapes’ value was explicitly represented, there was no significant stopping-induced devaluation [*t*(15) = 1.2, *p* = 0.25, *d* = 0.3]. Importantly, the difference between both conditions was significant, as predicted by our hypothesis [*t*(15) = 1.87, *p* = 0.041, one-sided, *d* = 0.54; Figure 3]. Hence, this within-subjects analysis of explicit learners confirmed the findings from the between group comparison, underscoring the fact that stopping-induced stimulus devaluation is indeed increased for explicit value representations.

### Control Analyses

Since six outliers were removed from the overall sample before parametric testing, we repeated our two main analyses of interest in non-parametric fashion, while including the entire sample of participants (i.e., including the outliers).

To test the STOPPING effect, we performed a Wilcoxon signed-rank test that compared the average bidding score for the shapes that were paired with stopping to the average bidding score for the other shapes. There was a significant effect (*z* = 2.36, *p* = 0.018, *r* = 0.292), showing again that stopping reduces stimulus value, and confirming the results from the ANOVA, as well as our prior studies (Wessel et al., 2014).

To test the KNOWLEDGE × STOPPING interaction, we computed the mean devaluation score for each participant (i.e., the difference between the average bid on the shapes that were paired with stopping and the average bid on the shapes that were not), and compared them between groups using a Mann-Whitney *U*-test. There was a significant effect (*z* = 2.2, *p* = 0.027, *r* = 0.273), showing that explicit learners showed stronger stopping-induced stimulus devaluation, confirming the results from the ANOVA.

We performed another control analysis to account for the fact that we changed the experimental parameters after the first 31 participants. To remind the reader, after 31 participants, we increased the trial numbers for the learning phase (and the stopping phase) because only five participants showed explicit knowledge of the differential values of the shapes in the learning phase. While this change of trial numbers was successful in increasing the relative proportion of explicit learners, it could have potentially influenced the results of our main hypothesis test (i.e., that explicit learners would show increased stopping-induced devaluation). Hence, we repeated the 2 × 2 × 4 ANOVA described above, but using only those participants that were collected *after* the adjustment of the trial numbers. Despite the considerably reduced power of this analysis [owing to a sample size reduction from 59 (22 explicit) to 33 (16 explicit)], our main results were replicated. The main effect of VALUE remained significant [*F*(3,93) = 2.8, *p* = 0.044, ${\text{η}}_{p}^{2}$ = 0.083], as did the main effect of STOPPING [*F*(1,31) = 5.87, *p* = 0.021, ${\text{η}}_{p}^{2}$ = 0.159]. The main effect of KNOWLEDGE remained not significant [*F*(1,31) = 0.29, *p* = 0.59, ${\text{η}}_{p}^{2}$ = 0.009]. As for the interactions, the KNOWLEDGE × VALUE interaction remained significant [*F*(3,93) = 3.13, *p* = 0.0295, ${\text{η}}_{p}^{2}$ = 0.092]. The KNOWLEDGE × STOPPING interaction remained significant as a two-sided trend [*F*(1,31) = 3.38, *p* = 0.076, ${\text{η}}_{p}^{2}$ = 0.098]. The VALUE × STOPPING interaction [*F*(3,93) = 0.05, *p* = 0.99, ${\text{η}}_{p}^{2}$ = 0.002] and the three-way interaction [*F*(3,93) = 1.76, *p* = 0.16, ${\text{η}}_{p}^{2}$ = 0.054] remained non-significant.

Finally, we repeated the main analysis of the paper while excluding eight participants that showed explicit knowledge of the stopping contingency. Just as in our previous report (Wessel et al., 2014), a small minority of participants developed an explicit representation of the fact that only some shapes were paired with stopping (as assessed in our post-experimental questionnaire, questions 2a and 2b). Since the results in these participants could potentially be affected by task demand characteristics, we excluded their data in the previous report, despite the fact that this exclusion did not affect the results. In the current study, these participants were not excluded, based on the same finding, i.e., based on the fact that their exclusion did not affect the results. The results of the ANOVA without the eight participants with explicit knowledge of the stopping contingency (three out of which were also explicit learners of the learning phase contingency, while five were not) were as follows: VALUE: *F*(3,156) = 8.77, *p* < 0.0001, ${\text{η}}_{p}^{2}$ = 0.1414; STOPPING: *F*(1,52) = 9.1, *p* = 0.004, ${\text{η}}_{p}^{2}$ = 0.149; KNOWLEDGE: *F*(1,52) = 1.97, *p* = 0.167, ${\text{η}}_{p}^{2}$ = 0.036; KNOWLEDGE × VALUE: *F*(3,156) = 3.72, *p* = 0.013, ${\text{η}}_{p}^{2}$ = 0.067; KNOWLEDGE × STOPPING: *F*(1,52) = 12.1, *p* = 0.001, ${\text{η}}_{p}^{2}$ = 0.189; VALUE × STOPPING: *F*(3,156) = 0.86, *p* = 0.46, ${\text{η}}_{p}^{2}$ = 0.016; VALUE × STOPPING × KNOWLEDGE: *F*(3,156) = 0.47, *p* = 0.71, ${\text{η}}_{p}^{2}$ = 0.009. Hence, the main effects of VALUE and STOPPING, as well as the two-way interactions of KNOWLEDGE × VALUE and KNOWLEDGE × STOPPING remained significant, while the main effect of KNOWLEDGE, as well as the VALUE × STOPPING interaction, and the three-way interaction remained non-significant. Since three outliers were removed from this analysis, we again repeated the main tests in non-parametric fashion, with the outliers remaining in the data. Again, these analyses confirmed the findings from the parametric analysis [*z* = 2.56, *p* = 0.01, *r* = 0.34, for the main effect of STOPPING, and *z* = 1.86, *p* = 0.031 (one-sided), *r* = 0.25, for the KNOWLEDGE × STOPPING interaction]. Taken together, these analyses show that excluding the participants with explicit knowledge of the stopping contingency did not affect our results.

### Exploration of Bidding Level Variance

In an auxiliary analysis, we tested whether the variance of the bidding levels for each shape was decreased in the explicit learner group compared to the implicit learner group. This would show that bidding variance is an objective indicator of different levels of knowledge about the task contingency (i.e., explicit learners would make more constant bids on the shapes). To test this, we used the same 2 × 2 × 4 ANOVA as for the main analysis (see Valuation Phase), with the exception that the DV was not the mean bid for each shape, but the variance of bidding level for each shape. For the 59 participants that comprised our main analysis, there was indeed a main effect of KNOWLEDGE on bidding variance [*F*(1,57) = 5.39, *p* = 0.024, ${\text{η}}_{p}^{2}$ = 0.086], showing that the explicit learners had significantly reduced dispersion in their bidding behavior, reflective of greater certainty with regards to the differential stimulus values. No other main effects or interactions were significant (all *p*-values > 0.3).

Additionally, we compared bidding variances within subjects in the explicit learner group. We averaged the bidding variances for the shapes that these participants explicitly named as being of specific value, and compared it to the bidding variances on the implicit shapes (i.e., those that were not explicitly associated with value). In line with the above results, this analysis showed that bidding variance was significantly reduced in shapes that were explicitly named to be of specific value, which was a significant two-sided trend [*t*(18) = 1.877, *p* = 0.077, *d* = 0.37]. These results further hint at bidding variance as a suitable indicator of explicit knowledge, at least in the context of value assayed in an auction such as the one in our current paradigm. It is furthermore possible (or even likely) that these trials account for the finding presented in the previous paragraph, i.e., that bidding variance differs between explicit and implicit learners only because of the shapes whose values were explicitly represented. However, it is hard to explicitly test this hypothesis in the current framework, as explicit learners differed with respect to the numbers and specific subsets of shapes whose values were explicitly represented. Hence, it is not possible to exclude the explicitly named shapes from the analysis for each explicit learner while still maintaining a factorial design with a valid between-subjects factor. Still, this analysis shows that bidding variance appears to be an indicator of bidding confidence, and directly related to the amount of explicit knowledge about stimulus value. However, since this was an exploratory analysis, these results have to be interpreted with caution, and further research is needed to explicitly elucidate the relationship between bidding variance and explicit knowledge of stimulus value.

### Analysis of Implicit Learners

In another exploratory data analysis, we investigated whether there was significant stopping-induced stimulus devaluation in our sample of implicit learners alone. We computed a 2 × 3 ANOVA that included only the factors VALUE and STOPPING (this is the same ANOVA that was done in Wessel et al., 2014). Just like the overall sample, the implicit learner group had a significant main effect of VALUE [*F*(3,126) = 3.31, *p* = 0.022, ${\text{η}}_{p}^{2}$ = 0.073]. However, there was no significant main effect of STOPPING [*F*(1,42) = 1.52, *p* = 0.22, ${\text{η}}_{p}^{2}$ = 0.035], despite a numerical reduction in bids for the shapes that were paired with stopping compared with shapes that were not, which was present in all four value conditions (Figure 2B). As in the overall sample, there was no STOPPING × VALUE interaction [*F*(3,126) = 0.48, *p* = 0.7, ${\text{η}}_{p}^{2}$ = 0.01].

**FIGURE 4:**
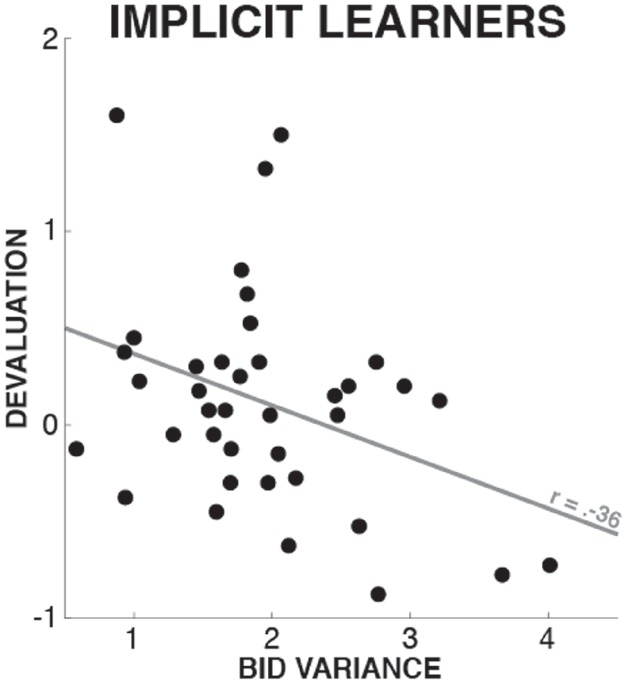
**Correlation between implicit learners’ bidding variance on the most and least valuable shapes and stopping-induced devaluation.** Since lower bid variance is a proxy of the degree of acquired task knowledge (see comparison of explicit vs. implicit learners in Section “Exploration of Bidding Level Variance”), this individual differences analysis suggests that implicit learners with higher degrees of acquired knowledge had a greater devaluation effect.

In a second exploratory analysis of the implicit learner group, we tested whether different degrees of acquired implicit knowledge were associated with different degrees of stopping-induced devaluation. To this end, we quantified the bidding variance for each participant in the implicit learner group, specifically for the shapes of highest ($4) and lowest ($0.5) value, to derive one compound measure of bidding variance that would best reflect the degree of acquired implicit knowledge about the differential values of the shapes. We selected the highest and lowest shapes because those were the stimuli whose differential values were the most obvious (this was reflected in the fact that any explicit knowledge about differential values in the *explicit* learner group always included the value of either the highest or lowest value stimuli). We then correlated this measurement with the participant’s devaluation scores (i.e., the difference in bidding level between the stopping and non-stopping shapes; see above). This analysis revealed a significant negative correlation across subjects (Pearson’s *r* = –0.36, *p* = 0.027; 5 out of 43 participants were removed from this analysis based on an outlier diagnostic based on a criterion of Cook’s distance >$\frac{4}{N}$). This correlation shows that implicit learners with less variance in their bids (i.e., who had greater amounts of acquired implicit knowledge) had a greater degree of stopping-induced stimulus devaluation (Figure 4).

## Discussion

We replicated Experiments 1 and 2 of Wessel et al. (2014) by showing again that action stopping reduces stimulus value. We also went further by showing that stopping-induced stimulus devaluation is increased in participants that have explicit knowledge about the relative values of rewarding stimuli, compared to participants whose stimulus-reward associations are fully implicit.

Our demonstration that stopping-induced devaluation is greater for explicit value representations could have strong practical importance. Most reward-related stimuli in realistic contexts are associated with explicit valuation; i.e., people are generally aware of their associated incentive value. The stimulus material in the current study closely mimics the nature of these real-life valuations. For example, most rewarding stimuli in real-life (e.g., a piece of cake or a cigarette) initially have very little associated reward value *per se*—similar to the shapes in our study. Over the lifespan, however, these stimuli become paired with rewarding outcomes, and hence, accumulate more and more explicit reward value, very similar to the shapes in our study. Our current findings imply that behavioral therapies using stopping could be successful in realistic contexts in which explicit reward associations have to be targeted for devaluation. They also suggest that reduced consumption of primary reinforcers after stopping or withholding a response to reinforcer-related stimuli could indeed be due to a reduction of value of these reinforcers, as previously speculated (Kiss et al., 2008; Veling et al., 2008, 2013; Houben et al., 2012a).

It is of note that while the group of participants with fully implicit value representations had a significantly reduced stopping-induced devaluation effect compared to the explicit group (in fact, stopping-induced devaluation in the implicit group was non-significant), there was still a significant main effect of value in the implicit group. Hence, these participants bid more for shapes that were worth more, despite not being able to verbalize any systematic value differences between shapes. This suggests that, even without an explicit value representation, they were not bidding randomly; and that the auction method was effective in capturing implicit value representations (see also Persaud et al., 2007). Furthermore, our exploratory data analyses indicated that even within this implicit learner group, the degree of acquired knowledge of the differential values was directly related to the degree of stopping-induced devaluation: participants with less variance in their bids (putatively reflecting a greater degree of acquired implicit knowledge) showed stronger stopping-induced stimulus devaluation. This is in line with the implicit learning literature, which shows that the emergence of explicit knowledge is a direct consequence of progressively increasing amounts of implicit knowledge (Seger, 1994; Frensch et al., 2003; Rose et al., 2010; Wessel et al., 2012). In our case, this would predict that the more knowledge implicit learners have acquired, the more similar their behavior (in this case, stopping-induced stimulus devaluation) should become to explicit learners, which was indeed the case. Taken together, these results further strengthen our initial assertion that more stable value representations are more susceptible to stopping-induced stimulus devaluation.

The question of why explicit value representations are more affected by stopping-induced devaluation comes down to the mechanism by which the action stopping procedure induces stimulus devaluation. Control experiments in our initial study (Wessel et al., 2014) argued against several possible explanations including infrequent signal detection, stimulus frequency, response-conflict, effort, or error processing, leaving the likely possibility that it was specifically a putatively broad suppressive process on stop trials that induced the value reduction. However, validating this theory requires assaying value representations trial-by-trial, and probably separating successful and failed stop trials; something that appears difficult with behavioral methods alone, and which awaits investigation with a technique such as fMRI. Future studies could also examine how long the stopping-induced devaluation lasts, and whether it generalizes to stimuli in a wider category.

In conclusion, while questions remain about the neural and psychological mechanisms by which stopping reduces stimulus value, whether it owes to successful or failed stop trials, and whether it is long-lasting and generalizes to other stimuli, the core effect is replicable. Moreover, we here show that it is particularly strong in those participants who have explicit value representations. This helps constrain the sorts of real world scenarios in which stopping-induced devaluation is likely to be effective. A natural next step would be to use an analogous procedure to test whether stimulus-devaluation can be achieved with stimuli that represent realistic rewards, such as cigarettes or cake. To facilitate such future investigations, we have provided documented code for running this experimental framework along with this paper.

## Author Contributions

JW and AA designed research. JW programmed the experiment. AT collected the data. JW analyzed the data. JW and AA wrote and edited the manuscript.

### Conflict of Interest Statement

The authors declare that the research was conducted in the absence of any commercial or financial relationships that could be construed as a potential conflict of interest.

## Footnotes

1 The fact that shapes were only rewarded according to the predetermined schedule on 80% of the trials actually reduces their associated value to 0.8 × ($0.5, 1, 2, 4); i.e., the actual values associated with the stimuli are $0.4, $0.8, $1.6, and $3.2, respectively. For simplicity reasons, we will continue to refer to the value steps according to the means of their reward distributions, i.e., $0.5, $1, $2, and $4.

2 Individual contrasts revealed the following significant pairings: High > Low (*p* < 0.0001), High > Medium Low (*p* = 0.0007), High > Medium High (*p* = 0.0014), Medium High > Low (*p* = 0.0274), Medium Low > low (*p* = 0.0479). The first three contrasts survive Bonferroni correction for multiple comparisons.

## References

[B1] Allom V. Mullan B. (2015). Two inhibitory control training interventions designed to improve eating behaviour and determine mechanisms of change. Appetite 89, 282–290. 10.1016/j.appet.2015.02.022 25725487

[B2] Becker G. M. Degroot M. H. Marschak J. (1964). Measuring utility by a single-response sequential method. Behav. Sci. 9, 226–232. 10.1002/bs.3830090304 5888778

[B3] Bowley C. Faricy C. Hegarty B. Johnstone S. J. Smith J. L. Kelly P. J. (2013). The effects of inhibitory control training on alcohol consumption, implicit alcohol-related cognitions and brain electrical activity. Int. J. Psychophysiol. 89, 342–348. 10.1016/j.ijpsycho.2013.04.011 23623953

[B4] Brainard D. H. (1997). The Psychophysics Toolbox. Spat. Vis. 10, 433–436. 10.1163/156856897X00357 9176952

[B5] Clark L. Averbeck B. Payer D. Sescousse G. Winstanley C. A. Xue G. (2013). Pathological choice: the neuroscience of gambling and gambling addiction. J. Neurosci. 33, 17617–17623. 10.1523/JNEUROSCI.3231-13.2013 24198353 PMC3858640

[B6] Doallo S. Raymond J. E. Shapiro K. L. Kiss M. Eimer M. Nobre A. C. (2012). Response inhibition results in the emotional devaluation of faces: neural correlates as revealed by fMRI. Soc. Cogn. Affect. Neurosci. 7, 649–659. 10.1093/scan/nsr031 21642353 PMC3427860

[B7] Fenske M. J. Raymond J. E. Kessler K. Westoby N. Tipper S. P. (2005). Attentional inhibition has social-emotional consequences for unfamiliar faces. Psychol. Sci. 16, 753–758. 10.1111/j.1467-9280.2005.01609.x 16181435

[B8] Ferrey A. E. Frischen A. Fenske M. J. (2012). Hot or not: response inhibition reduces the hedonic value and motivational incentive of sexual stimuli. Front. Psychol. 3:575. 10.3389/fpsyg.2012.00575 23272002 PMC3530044

[B9] Frensch P. A. Haider H. Rünger D. Neugebauer U. Voigt S. Werg J. (2003). “The route from implicit learning to verbal expression of what has been learned,” in Attention and Implicit Learning, ed. Jiménez L. (Amsterdam: John Benjamins Publishing Company), 335–366.

[B10] Frischen A. Ferrey A. E. Burt D. H. Pistchik M. Fenske M. J. (2012). The affective consequences of cognitive inhibition: devaluation or neutralization? J. Exp. Psychol. Hum. Percept. Perform. 38, 169–179. 10.1037/a0025981 22022896

[B11] Hogarth L. Dickinson A. Duka T. (2010). The associative basis of cue-elicited drug taking in humans. Psychopharmacology (Berl.) 208, 337–351. 10.1007/s00213-009-1735-9 19960187

[B12] Houben K. (2011). Overcoming the urge to splurge: influencing eating behavior by manipulating inhibitory control. J. Behav. Ther. Exp. Psychiatry 42, 384–388. 10.1016/j.jbtep.2011.02.008 21450264

[B13] Houben K. Havermans R. C. Nederkoorn C. Jansen A. (2012a). Beer a no-go: learning to stop responding to alcohol cues reduces alcohol intake via reduced affective associations rather than increased response inhibition. Addiction 107, 1280–1287. 10.1111/j.1360-0443.2012.03827.x 22296168

[B14] Houben K. Nederkoorn C. Jansen A. (2012b). Too tempting to resist? Past success at weight control rather than dietary restraint determines exposure-induced disinhibited eating. Appetite 59, 550–555. 10.1016/j.appet.2012.07.004 22796949

[B15] Houben K. Jansen A. (2011). Training inhibitory control. A recipe for resisting sweet temptations. Appetite 56, 345–349. 10.1016/j.appet.2010.12.017 21185896

[B16] Houben K. Nederkoorn C. Wiers R. W. Jansen A. (2011). Resisting temptation: decreasing alcohol-related affect and drinking behavior by training response inhibition. Drug Alcohol Depend 116, 132–136. 10.1016/j.drugalcdep.2010.12.011 21288663

[B17] Jones A. Field M. (2013). The effects of cue-specific inhibition training on alcohol consumption in heavy social drinkers. Exp. Clin. Psychopharmacol. 21, 8–16. 10.1037/a0030683 23181512

[B18] Kiss M. Raymond J. E. Westoby N. Nobre A. C. Eimer M. (2008). Response inhibition is linked to emotional devaluation: behavioural and electrophysiological evidence. Front. Hum. Neurosci. 2:13. 10.3389/neuro.09.013.2008 18958213 PMC2572209

[B19] Lawrence N. S. Verbruggen F. Morrison S. Adams R. C. Chambers C. D. (2015). Stopping to food can reduce intake. Effects of stimulus-specificity and individual differences in dietary restraint. Appetite 85, 91–103. 10.1016/j.appet.2014.11.006 25447023 PMC4286116

[B20] Logan G. D. Cowan W. B. Davis K. A. (1984). On the ability to inhibit simple and choice reaction-time responses—a model and a method. J. Exp. Psychol. Hum. Percept. Perform. 10, 276–291. 10.1037//0096-1523.10.2.276 6232345

[B21] Marteau T. M. Hollands G. J. Fletcher P. C. (2012). Changing human behavior to prevent disease: the importance of targeting automatic processes. Science 337, 1492–1495. 10.1126/science.1226918 22997327

[B22] Nederkoorn C. Coelho J. S. Guerrieri R. Houben K. Jansen A. (2012). Specificity of the failure to inhibit responses in overweight children. Appetite 59, 409–413. 10.1016/j.appet.2012.05.028 22664299

[B23] Nederkoorn C. Houben K. Hofmann W. Roefs A. Jansen A. (2010). Control yourself or just eat what you like? Weight gain over a year is predicted by an interactive effect of response inhibition and implicit preference for snack foods. Health Psychol. 29, 389–393. 10.1037/a0019921 20658826

[B24] Persaud N. Mcleod P. Cowey A. (2007). Post-decision wagering objectively measures awareness. Nat. Neurosci. 10, 257–261. 10.1038/nn1840 17237774

[B25] Raymond J. E. Fenske M. J. Westoby N. (2005). Emotional devaluation of distracting patterns and faces: a consequence of attentional inhibition during visual search? J. Exp. Psychol. Hum. Percept. Perform. 31, 1404–1415. 10.1037/0096-1523.31.6.1404 16366798

[B26] Root D. H. Fabbricatore A. T. Barker D. J. Ma S. Pawlak A. P. West M. O. (2009). Evidence for habitual and goal-directed behavior following devaluation of cocaine: a multifaceted interpretation of relapse. PLoS ONE 4:e7170. 10.1371/journal.pone.0007170 19779607 PMC2744871

[B27] Rose M. Haider H. Buchel C. (2010). The emergence of explicit memory during learning. Cereb. Cortex 20, 2787–2797. 10.1093/cercor/bhq025 20194687

[B28] Rothemund Y. Preuschhof C. Bohner G. Bauknecht H. C. Klingebiel R. Flor H. (2007). Differential activation of the dorsal striatum by high-calorie visual food stimuli in obese individuals. Neuroimage 37, 410–421. 10.1016/j.neuroimage.2007.05.008 17566768

[B29] Rupprecht L. E. Smith T. T. Schassburger R. L. Buffalari D. M. Sved A. F. Donny E. C. (2015). Behavioral mechanisms underlying nicotine reinforcement. Curr. Top. Behav. Neurosci. 24, 19–53. 10.1007/978-3-319-13482-6_2 25638333 PMC4536896

[B30] Schonberg T. Bakkour A. Hover A. M. Mumford J. A. Nagar L. Perez J. (2014). Changing value through cued approach: an automatic mechanism of behavior change. Nat. Neurosci. 17, 625–U195. 10.1038/nn.3673 24609465 PMC4041518

[B31] Schonberg T. Fox C. R. Poldrack R. A. (2011). Mind the gap: bridging economic and naturalistic risk-taking with cognitive neuroscience. Trends Cogn. Sci. 15, 11–19. 10.1016/j.tics.2010.10.002 21130018 PMC3014440

[B32] Seger C. A. (1994). Implicit learning. Psychol. Bull. 115, 163–196. 10.1037/0033-2909.115.2.163 8165269

[B33] Sescousse G. Barbalat G. Domenech P. Dreher J. C. (2013). Imbalance in the sensitivity to different types of rewards in pathological gambling. Brain 136, 2527–2538. 10.1093/brain/awt126 23757765

[B34] Veling H. Aarts H. Stroebe W. (2013). Stop signals decrease choices for palatable foods through decreased food evaluation. Front. Psychol. 4:875. 10.3389/fpsyg.2013.00875 24324451 PMC3840792

[B35] Veling H. Holland R. W. Van Knippenberg A. (2008). When approach motivation and behavioral inhibition collide: behavior regulation through stimulus devaluation. J. Exp. Soc. Psychol. 44, 1013–1019. 10.1016/j.jesp.2008.03.004

[B36] Verbruggen F. Logan G. D. (2009). Models of response inhibition in the stop-signal and stop-change paradigms. Neurosci. Biobehav. Rev. 33, 647–661. 10.1016/j.neubiorev.2008.08.014 18822313 PMC2696813

[B37] Wessel J. R. Haider H. Rose M. (2012). The transition from implicit to explicit representations in incidental learning situations: more evidence from high-frequency EEG coupling. Exp. Brain Res. 217, 153–162. 10.1007/s00221-011-2982-7 22186962

[B38] Wessel J. R. O’Doherty J. P. Berkebile M. M. Linderman D. Aron A. R. (2014). Stimulus devaluation induced by stopping action. J. Exp. Psychol. Gen. 143, 2316–2329. 10.1037/xge0000022 25313953 PMC4244281

[B39] Wisotsky W. Swencionis C. (2003). Cognitive-behavioral approaches in the management of obesity. Adolesc. Med. 14, 37–48. 12529189

